# MicroRNAs and their role in heart transplantation insights into rejection mechanisms: A narrative review

**DOI:** 10.1016/j.jhlto.2026.100496

**Published:** 2026-01-20

**Authors:** Rossella Loria, Antonio Giuseppe Bianculli, Paola Giustiniani, Maria Troiano, Marco Andreani, Giorgia Grutter

**Affiliations:** a Cellular Network and Molecular Therapeutic Target Unit, IRCCS Regina Elena National Cancer Institute, Rome, Italy; b Laboratory of Transplant Immunogenetics, Department of Hematology/Oncology, Cell and Gene Therapy, Bambino Gesù Children's Hospital, IRCCS, Rome, Italy; c Interventional Cardiology of Congenital Heart Diseases, Bambino Gesù Children’s Hospital, IRCCS, Rome, Italy

**Keywords:** Heart transplantation, MiRNAs, Graft failure, Rejection, Biopsy

## Abstract

Heart transplantation is the definitive treatment for patients with advanced heart failure and refractory symptoms. However, allograft rejection—both acute and chronic—remains a major cause of morbidity, leading to graft dysfunction and failure. Traditionally, endomyocardial biopsy (EMB) has been the standard method for screening allograft rejection. MicroRNAs (miRNAs) are small, non-coding RNA sequences that regulate gene expression by binding to the 3' untranslated regions of complementary mRNA transcripts. This review explores the potential of miRNAs as biomarkers for detecting allograft rejection in heart transplant recipients. MiRNAs may serve as non-invasive “liquid biopsies,” providing a novel approach to monitor and manage post-transplant patients.

## Background

Heart transplantation is a therapeutic option for patients with end-stage heart failure. However, post-transplant complications, particularly acute and chronic rejection, remain significant challenges. Early detection of rejection is critical to prevent graft dysfunction and enhance long-term survival. Endomyocardial biopsy (EMB) is the most widely used and well-established method for diagnosing rejection, considered the gold standard for detecting acute cellular rejection (ACR) and, to some extent, antibody-mediated rejection (AMR) EMB involves inserting a catheter-based bioptome through a central vein, typically the right internal jugular vein, to obtain myocardial tissue samples for histopathological analysis.^1^ These samples are examined using the International Society for Heart and Lung Transplantation grading system, which classifies rejection severity based on cellular infiltration and tissue damage.^2^ However, despite its diagnostic utility, EMB has limitations, including invasiveness, risks, and sampling errors.^3^ As a result, there has been growing interest in developing non-invasive strategies for rejection surveillance, though these methods still face challenges in terms of diagnostic accuracy and reliability.^4^ In recent years, MicroRNAs (miRNAs) have emerged as important regulators in heart transplantation, influencing both favorable and unfavorable prognostic outcomes.^5^ This review examines the role of miRNAs in transplantation mechanisms.

## MicroRNAs: Biogenesis and functional roles

miRNAs are small, non-coding RNA molecules that play a critical role in regulating gene expression at the post-transcriptional level. By binding to target messenger RNAs (mRNAs), miRNAs can either inhibit translation or promote mRNA degradation.^6^ Over the past 2 decades, miRNAs have emerged as essential regulators of numerous biological processes, including development, differentiation, apoptosis, and immune responses.^7^

In the field of heart transplantation, miRNAs have attracted significant interest for their potential as non-invasive biomarkers and therapeutic targets. Their involvement in key pathways, including graft rejection, fibrosis, angiogenesis, and ischemia-reperfusion injury, underscores their promise in improving graft survival and overall patient outcomes.^5^

### Biogenesis of miRNAs

The generation of mature miRNAs is a multi-step process that is tightly controlled both in the nucleus and the cytoplasm.

**Figure 1:**
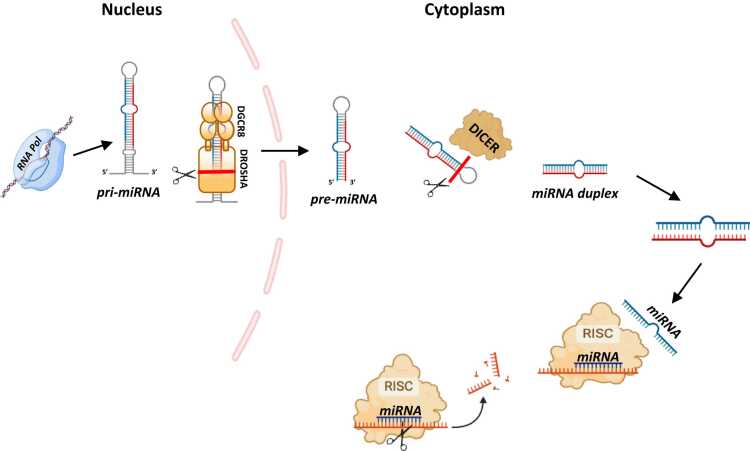
*Biogenesis of microRNAs (miRNAs).* The process begins in the nucleus, where miRNA genes are transcribed by RNA polymerase II (or III) into primary miRNAs (pri-miRNAs). These pri-miRNAs are processed by the microprocessor complex, consisting of Drosha and DGCR8, into precursor miRNAs (pre-miRNAs), which are then exported to the cytoplasm by Exportin-5. In the cytoplasm, the enzyme Dicer further processes pre-miRNAs into mature miRNA duplexes. One strand of the miRNA duplex is incorporated into the Argonaute-containing RISC, while the other strand is degraded. The mature miRNA then guides RISC to target mRNA, leading to translational repression or mRNA degradation. Key proteins and cellular compartments involved in each step are indicated.

miRNAs are initially transcribed by RNA polymerase II or III as long precursor molecules called primary miRNAs (pri-miRNAs) that possess a characteristic hairpin structure^6^, ^8^ (Figure 1). Some pri-miRNAs are transcribed independently by intergenic promoters, while others share a common promoter with their host genes or are co-transcribed as polycistronic units.^9^ Pri-miRNAs are typically capped and polyadenylated, preparing them for further processing.

Within the nucleus, the pri-miRNAs are processed by the microprocessor complex, which includes the RNase III enzyme Drosha and its cofactor DGCR8, forming a pre-miRNA of approximately 70 nucleotides in length (Figure 1).

The pre-miRNAs are then exported to the cytoplasm by Exportin-5, a nuclear transport protein. In the cytoplasm, the RNase III enzyme Dicer processes the pre-miRNA to generate a double-stranded miRNA duplex of approximately 22 nucleotides. From this duplex, one strand (the guide strand) is selectively loaded into the RNA-induced silencing complex (RISC), while the other (the passenger strand) is usually degraded. Once incorporated into RISC, the guide strand associates with Argonaute (AGO) proteins, enabling it to recognize and bind to complementary sequences within the 3' untranslated region of target mRNAs. This binding results in gene regulation through either inhibition of translation or promotion of mRNA degradation^6^, ^10^ (Figure 1).

Beyond this canonical pathway, several non-canonical biogenesis mechanisms have been identified.^11^ Additionally, miRNAs can also be secreted into the extracellular environment through various mechanisms, such as exosomes, microvesicles, or association with RNA-binding proteins like Argonaute 2 or high-density lipoproteins. This secretion enables miRNAs to function as intercellular signaling molecules, mediating communication between cells and influencing physiological and pathological processes, including immune response.^12^

### Functional roles and quantification of miRNAs in heart transplantation

miRNAs regulate gene expression and contribute to critical biological processes, including development, differentiation, cell cycle control, apoptosis, and immune modulation.^7^, ^12^ Increasing evidence highlights their relevance in heart transplantation.

#### Non-invasive biomarkers

Circulating miRNAs demonstrate high stability in biofluids and transplant-specific expression patterns, making them promising candidates for noninvasive rejection monitoring. Their measurement may reduce reliance on routine EMB while enabling more frequent assessment of graft status.

#### Graft function and immune regulation

miRNAs modulate pathways involved in inflammation, angiogenesis, and ischemia-reperfusion injury—processes central to graft performance and post-transplant immune responses. These regulatory roles provide mechanistic insight into graft dysfunction and may identify novel therapeutic targets.

#### Therapeutic relevance

Targeting dysregulated miRNAs offers potential to attenuate rejection, limit fibrosis, and enhance myocardial repair, thereby complementing standard immunosuppression.

Robust quantification is essential to translate miRNAs into clinical practice.^12^, ^13^ Droplet digital PCR (ddPCR) enables absolute quantification without standard curves and is particularly suited for low-abundance targets or limited/degraded samples.^14^, ^15^ Partitioning into thousands of reactions and fluorescence-based detection provide high analytical sensitivity, precision, and reproducibility, surpassing qPCR performance.^16^

Detection of circulating miRNAs by ddPCR supports liquid biopsy applications. Tracking dynamic expression changes may improve treatment stratification and enable personalized post-transplant management.

## Invasive and non-invasive tests to detect rejection after heart transplantation

### Invasive tests to detect rejection

EMB remains the standard method for diagnosing rejection, but its limitations are well recognized. As an invasive procedure, it carries risks, including bleeding, infection, arrhythmias, cardiac perforation, and tricuspid valve injury.^17^ EMB is also subject to sampling error due to the often-patchy distribution of rejection, and it provides only a single time-point assessment, requiring repeated procedures for longitudinal monitoring.

These challenges have driven growing interest in complementary noninvasive strategies capable of improving diagnostic accuracy while reducing biopsy burden.

Molecular Microscopy Diagnostic System incorporates RNA expression profiling into biopsy assessment and offers enhanced sensitivity for detecting rejection beyond conventional histopathology.^18^ By capturing molecular signatures of myocardial injury and immune activation, Molecular Microscopy Diagnostic System provides a more comprehensive evaluation of graft status and may support improved clinical decision-making.

As molecular diagnostics continue to evolve, their integration into routine surveillance has the potential to refine rejection detection and reduce reliance on EMB in heart transplant recipients.

### Non-invasive tests to detect rejection

Several noninvasive approaches have been developed to complement EMB. Gene expression profiling (GEP) assesses immune-related transcripts in peripheral blood. The AlloMap test is an approved GEP assay used to stratify rejection risk and reduce routine biopsy frequency in stable patients.^18^ Although its negative predictive value is high, GEP is less effective for confirming rejection, limiting its use as a standalone diagnostic tool.

Donor-derived cell-free DNA (dd-cfDNA) represents another promising biomarker. Myocardial injury during rejection leads to increased release of donor DNA fragments into the recipient’s circulation. Elevated dd-cfDNA levels correlate with both ACR and AMR, offering a sensitive and non-invasive method for rejection surveillance.^19^ Ongoing studies aim to refine thresholds and standardize clinical implementation.

Advanced cardiac imaging techniques are also under evaluation. Cardiac magnetic resonance (CMR), particularly with T2-weighted imaging and T1 mapping, enables detection of myocardial edema and fibrosis linked to rejection. Similarly, strain echocardiography can identify subclinical ventricular dysfunction prior to overt histological changes.^20^ While these modalities provide valuable functional and tissue information, further validation is required before routine adoption in transplant monitoring.

## Role of miRNAs in graft rejection

**Figure 2:**
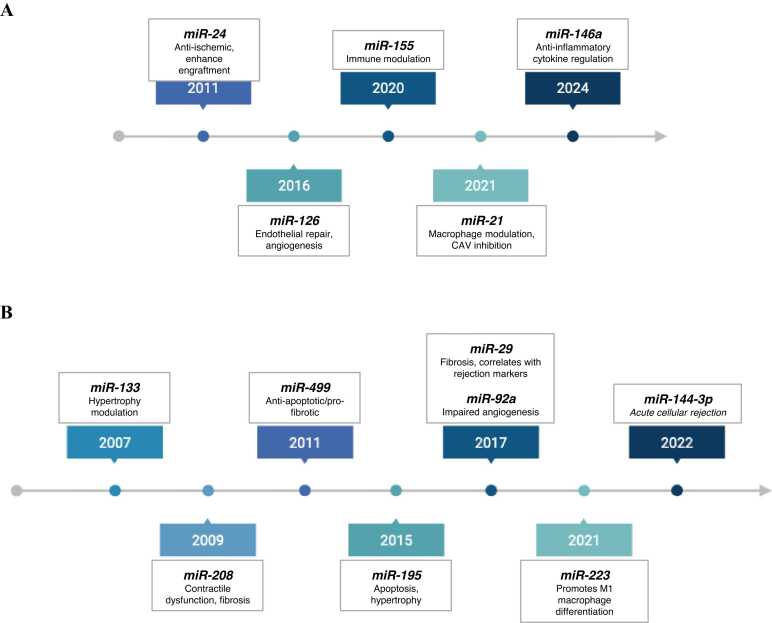
Timeline of miRNAs associated with (A) favorable and (B) unfavorable/ambiguos prognosis.

MiRNAs play a crucial role in gene expression regulation and are increasingly studied as biomarkers in heart transplantation. Some miRNAs are associated with favorable outcomes, promoting graft tolerance and survival, while others correlate with unfavorable outcomes, contributing to rejection or graft dysfunction. Additionally, some miRNAs exhibit ambiguous or controversial roles, with conflicting evidence regarding their influence on post-transplant immunological and fibrotic processes. Understanding these roles is key to developing targeted therapeutic strategies and improving the management of heart transplant recipients (Table 1, Figure 2).

**Table 1:** Summary of Key miRNAs in Heart Transplantation

miRNA	Prognosis	Function	Evidence type	Reference
miR-21	Favorable	Macrophage modulation, CAV inhibition	Animal (mouse model)	Ref.^21^
miR-126	Favorable	Endothelial repair, angiogenesis	Animal	Ref.^22^
miR-24	Favorable	Anti-ischemic, enhances engraftment	Animal	Ref.^23^, ^24^
miR-146a	Favorable	Anti-inflammatory cytokine regulation	Animal	Ref.^25^
miR-155	Favorable	Immune modulation	Human	Ref.^26^, ^27^
miR-144-3p	Unfavorable	Biomarker of acute cellular rejection; associated with rejection severity	Human	Ref.^28^, ^29^
miR-29	Unfavorable	Fibrosis, correlates with rejection markers	Human	Ref.^30^
miR-92a	Unfavorable	Impaired angiogenesis	Human	Ref.^28^, ^31^
miR-195	Unfavorable	Apoptosis, hypertrophy	Animal	Ref.^32^
miR-223	Unfavorable	Promotes M1 macrophage differentiation	Human + Animal	Ref.^27^
miR-208	Ambiguous	Contractile dysfunction, fibrosis	Human + Animal	Ref.^33^
miR-133	Ambiguous	Hypertrophy modulation	Human + Animal	Ref.^33^, ^34^, ^35^
miR-499	Ambiguous	Anti-apoptotic/pro-fibrotic	Human + Animal	Ref.^33^, ^34^, ^35^

Abbreviations: CAV, cardiac allograft vasculopathy; miRNAs, MicroRNAs.

Selection criteria for miRNAs included in this review: the literature search was performed between July 2024 and March 2025 in PubMed, Scopus, and Web of Science databases, covering studies published from 2007 to 2025. The keywords used included: microRNA, heart transplantation, graft rejection, CAV, and biomarkers. Only peer-reviewed articles written in English were considered. Eligible studies included human data and relevant preclinical animal models that reproduced immunological and fibrotic mechanisms associated with graft rejection or dysfunction. Priority was given to miRNAs for which functional evidence, correlation with clinical outcomes, or mechanistic involvement in cardiac transplant pathology was reported. miRNAs were categorized based on the predominant evidence in the literature as favorable (associated with tolerance or improved graft function), unfavorable (associated with rejection, fibrosis, or graft dysfunction), or ambiguous/controversial when conflicting results were reported. The final selection included miRNAs consistently described across multiple sources or demonstrating translational potential, as summarized in Table 1 and Figure 2.

### miRNAs associated with favorable prognosis

#### miR-21 (Preclinical, murine model)

miR-21 is one of the most extensively studied miRNAs in the context of cardiovascular diseases and heart transplantation. A study conducted by Usuelli et al examined the role of miR-21 in cardiac allograft vasculopathy (CAV) using a murine model. They found that miR-21 was significantly expressed and primarily localized to macrophages in heart samples from both the murine model and cardiac-transplanted patients with CAV. Targeting miR-21 in macrophages resulted in indefinite cardiac allograft survival and abrogated CAV. This suggests that miR-21 plays a crucial role in the development of CAV and that targeting it could improve transplant outcomes. These studies highlight the potential of miR-21 as a therapeutic target in cardiac transplantation, offering a promising avenue for improving graft survival and function.^21^

#### miR-126 (Preclinical, endothelial cells in murine model)

miR-126 is well known for its role in endothelial protection and angiogenesis. Evidence summarized by Mormile indicates that altered miR-126 expression may contribute to endothelial injury, a process that is also central in CAV. Although most data derive from non-transplant settings, these observations provide a strong biological rationale for the investigation of miR-126 in graft adaptation and vascular integrity.^22^

#### miR-24 (Preclinical, murine ischemia-reperfusion model)

miR-24 has been implicated in protecting the myocardium from ischemia-reperfusion injury, a mechanism relevant to heart transplantation. Hu et al demonstrated that a microRNA-based prosurvival cocktail containing miR-24 enhanced engraftment and function of transplanted cardiac progenitor cells.^23^ Additional studies support a role of miR-24 in promoting angiogenesis and tissue repair during ischemic stress, further suggesting its therapeutic potential after transplantation.^24^

#### miR-146a (Animal plasma samples; In vitro functional assays)

miR-146a is a negative regulator of NF-κB–dependent inflammatory signaling and contributes to the control of innate and adaptive immune responses in preclinical models. Circulating non-coding RNAs involved in immune pathways, including miR-146a, are under evaluation as non-invasive biomarkers for rejection in heart transplant recipients, although clinical validation is still ongoing.^25^ Overall, miR-146a is considered a promising protective miRNA and may serve as a therapeutic target to promote tolerance after heart transplantation.

#### miR-155 (Clinical: Heart transplant recipients)

Although miR-155 is generally considered pro-inflammatory, some studies have highlighted that its controlled expression may contribute to immune tolerance in heart transplantation. A study by Van Aelst et al (2016) evaluated miR-155 expression and its dual role in heart transplant recipients. Researchers collected serum samples from patients with and without rejection episodes, analyzing miR-155 expression through quantitative PCR. The results demonstrated elevated miR-155 levels in patients without rejection, suggesting a possible role in immune regulation.^26^ In addition, Novák et al (2021) reviewed the role of miRNAs as theranostic biomarkers in cardiac allograft transplantation, bridging findings from murine models to clinical applications. The study highlighted the diagnostic and prognostic potential of miR-155 in detecting graft rejection and promoting immune tolerance. It also explored the feasibility of using miRNAs, including miR-155, as therapeutic targets to improve transplant outcomes.^27^ These findings support the integration of microRNA-based strategies for enhancing transplant survival.

### miRNAs associated with unfavorable prognosis

#### miR-144-3p (Clinical: Human plasma samples; In vitro functional assays)

In contrast, recent clinical transcriptomic studies revealed miR-144-3p as a strong diagnostic biomarker for ACR in adult heart transplant recipients, demonstrating high accuracy in distinguishing rejection severity. Multicenter liquid-biopsy studies (GRAfT consortium) identified panels of circulating miRNAs capable of noninvasively detecting both ACR and AMR with high diagnostic performance.^28^, ^29^

#### miR-29 (Clinical: Patient serum correlation with rejection markers)

miR-29 has been implicated in the process of cardiac fibrosis, a critical aspect of heart transplant rejection. A study comparing the correlations between miR-29 and rejection grade revealed that the level of miR-29 positively correlated with cTnI, NT-proBNP, white blood cell counts and negatively with lymphocyte counts (all *p* < 0.001). The authors concluded that miR-29 could serve as a promising predictor of the risk of heart transplant rejection, providing valuable insights into transplant management.^30^

#### miR-92a (Clinical: Circulating miRNAs from heart transplant recipients)

Elevated expression of miR-92a has been associated with endothelial dysfunction and impaired angiogenic capacity. Although this evidence derives primarily from chronic kidney disease models (Shang et al), these mechanisms may also play a role in the compromised vascular repair observed in transplanted organs, potentially contributing to an increased risk of graft injury.^31^

In the context of heart transplantation, Shah et al conducted a multicenter investigation within the Genomic Research Alliance for Transplantation (GRAfT). Using small RNA sequencing of plasma samples obtained at the time of EMB, the authors compared patients without rejection to those with ACR or AMR. Among several novel circulating miRNAs, miR-92a demonstrated strong performance as a noninvasive biomarker for the diagnosis of acute rejection after heart transplantation.^28^

#### miR-195 (Preclinical: Murine cardiac hypertrophy and apoptosis models)

miR-195 has been associated with myocardial hypertrophy and apoptosis. Studies have shown that increased expression of miR-195 in transplanted hearts can lead to myocardial dysfunction and contribute to graft failure. An analysis of endomyocardial biopsies from heart transplant recipients revealed that high miR-195 levels were associated with increased expression of pro-apoptotic genes, such as BAX and Caspase-3. Additionally, experiments on animal models demonstrated that inhibiting miR-195 led to improved contractile function and reduced cardiomyocyte apoptosis. These findings suggest that miR-195 could be a therapeutic target, though further studies are needed to confirm its efficacy for improving cardiac graft function and reducing the risk of post-transplant heart failure.^32^

#### miR-223 (Clinical + preclinical: Human rejection samples and murine models)

miR-223 plays a significant role in regulating the immune response, and its upregulation has been associated with increased immune cell infiltration in heart transplant grafts, contributing to acute rejection. A clinical study on patients experiencing acute rejection found significantly higher levels of miR-223 in comparison to patients with stable graft function. In vitro experiments demonstrated that miR-223 regulates M1 macrophage differentiation, promoting a pro-inflammatory phenotype. Additionally, experiments in murine models showed that inhibiting miR-223 reduces inflammation and prolongs graft survival. These findings suggest that miR-223 could be a useful biomarker for monitoring acute rejection and a potential target for immunomodulatory therapies to improve post-transplant outcomes.^27^

#### miR-208 (Clinical + preclinical: Human biopsies and murine fibrosis models)

miR-208 is involved in regulating the expression of cardiac contractile proteins. Although it is associated with physiological adaptation, its post-transplant upregulation may contribute to maladaptive responses that negatively impact graft prognosis. A study analyzing endomyocardial biopsies from heart transplant recipients revealed elevated miR-208 levels in cases of chronic rejection. Murine model experiments showed that miR-208 modulates myosin light chain expression, contributing to pathological graft remodeling. Functional studies on human cardiomyocytes indicated that miR-208 overexpression is linked to reduced contractility and increased interstitial fibrosis. These findings suggest that controlling miR-208 expression could be a potential strategy to improve post-transplant cardiac function.^33^

### miRNAs with ambiguous or controversial roles

miR-133, miR-499, and miR-208 exhibit conflicting roles in cardiac pathophysiology and, by extension, may exert context-dependent effects in the transplanted heart, showing both protective and pathogenic effects depending on experimental conditions and timing. miR-133 has shown antifibrotic effects in human biopsies but also contributes to hypertrophy in animal models. miR-499 is associated with anti-apoptotic properties, yet its overexpression may promote fibrosis. miR-208 has a dual role: regulating cardiac contractility in adaptation but also implicated in chronic graft remodeling. These contradictory findings underscore the need for contextual interpretation and further validation in standardized human studies.^33^, ^34^, ^35^

## Distinct miRNA patterns for differentiating infection from inflammation

Emerging data support the role of distinct miRNA profiles in distinguishing infection-related inflammation from non-rejection inflammatory processes, including autoimmune and sterile inflammatory responses. Distinct miRNA patterns have been shown to differentiate between infection and non-rejection-related inflammation, such as that seen in autoimmune diseases or sterile inflammation. For example, specific miRNAs, like miR-146a, miR-155, and miR-21, have been linked to inflammation and immune responses in infection, while others, like miR-223, are more indicative of sterile inflammation.^36^ Identifying these miRNA signatures can provide a more accurate method to distinguish between infection and other forms of inflammation, aiding in diagnosis and the development of therapeutic strategies.^37^ Such patterns may also be instrumental in differentiating between rejection and non-rejection inflammatory responses, a critical aspect in transplant medicine.^38^

## Conclusion

Although the clinical translation of miRNA-based technologies presents challenges, the landscape is increasingly encouraging. Advances in extraction protocols, normalization strategies, and analytical tools (particularly ddPCR and next-generation sequencing) are steadily reducing variability and improving reproducibility across centers. At the same time, the biological complexity of miRNAs, once viewed as an obstacle, is now recognized as a unique strength. Their capacity to mirror simultaneous alterations across multiple biological pathways makes them exceptionally informative biomarkers, capable of capturing the full spectrum of immune activation, tissue injury, and graft adaptation in ways that single-analyte markers cannot. As multicenter initiatives and biobank-linked longitudinal datasets grow, the interpretation of miRNA signatures is becoming more robust. Machine-learning algorithms, systems-biology models, and integrative multi-omics approaches are increasingly applied to miRNA panels, helping extricate the influence of comorbidities, immunosuppressive regimens, and individual patient variability and accelerating the identification of clinically informative signatures with strong discriminatory capacity.

Clinical validation efforts are also progressing. Several miRNAs have already demonstrated diagnostic performance comparable to established modalities in small and medium-sized cohorts, and larger validation trials are underway. Importantly, a clear conceptual framework now guides the integration of miRNAs into clinical practice. Rather than replacing established tools, miRNAs are poised to complement EMB, gene-expression profiling, and dd-cfDNA by enhancing sensitivity for early rejection, refining risk stratification, and potentially reducing biopsy frequency. Their alignment with the current trajectory of precision transplant medicine makes them particularly suited for future adoption.

Equally promising is the broader scientific environment, which is increasingly primed for molecular biomarkers. The successful clinical implementation of gene-expression profiling and dd-cfDNA illustrates that transplant medicine embraces novel technologies when they demonstrate clear clinical utility. Emerging miRNA platforms, backed by faster turnaround times, more cost-effective workflows, and advanced computational interpretation, are expected to follow a similar path. With sustained multidisciplinary collaboration, uniting cardiologists, transplant immunologists, molecular biologists, data scientists, and bioengineers, the remaining translational barriers are likely to diminish rapidly.

In conclusion, microRNAs are emerging as powerful molecular tools with the potential to transform the management of heart transplant recipients. A growing body of evidence highlights the protective and immunoregulatory roles of miRNAs such as miR-21, miR-126, miR-24, miR-146a, and miR-155, which collectively contribute to endothelial repair, graft adaptation, and modulation of post-transplant inflammation. Conversely, miRNAs including miR-29, miR-92a, miR-195, miR-223, and miR-208 are closely associated with adverse processes such as fibrosis, endothelial dysfunction, and heightened alloimmune activation, making them valuable markers for identifying patients at increased risk of rejection. A third group, comprising miR-133, miR-499, and context-dependent forms of miR-208, exhibits variable roles under different physiological and pathological conditions, reflecting the intricate regulatory networks through which miRNAs operate in the transplanted heart.

Taken together, expanding biological insights, rapid technological progress, and growing clinical readiness paint a highly optimistic picture for the future of miRNA translation in heart transplantation. Although further validation and refinement are needed, none of the remaining challenges appear insurmountable. As research continues to advance, miRNA-based diagnostics and therapeutics are poised to become integral components of precision transplant medicine, offering new avenues to enhance rejection surveillance, personalize immunosuppression, and ultimately improve long-term graft survival and patient outcomes.

## Conflicts of Interest statement

The authors declare that they have no known competing financial interests or personal relationships that could have appeared to influence the work reported in this paper.
